# Trade-offs in survival strategies: root trait differentiation of trees and shrubs on the Jingpo Lake lava platform

**DOI:** 10.1515/biol-2025-1267

**Published:** 2026-02-11

**Authors:** Liying Xu, Wei Peng, Xiang Li, Xinxin Zhang, Xinmei Li, Jiarui Zhang, Yixin Sun, Guangrong Gao, Dounan Liu, Yueqi Zhao

**Affiliations:** School of Life Science and Technology, Mudanjiang Normal College, Mudanjiang 157011, Heilongjiang, P.R. China; School of Chemistry and Chemical Engineering, Mudanjiang Normal College, Mudanjiang 157011, Heilongjiang, P.R. China

**Keywords:** heterogeneous habitats, vegetation restoration, survival strategies, root traits

## Abstract

To investigate the adaptive strategies of root functional traits in woody plants of different life forms on the Jingpo Lake lava platform towards heterogeneous habitats and provide reference for vegetation restoration strategies on lava platforms, this study examined 16 common woody plant species (12 trees and four shrubs) on the Jingpo Lake lava platform and measured the morphology and chemical properties of their primary to tertiary roots to investigate differences in root functional traits among plants of varying life forms. Trees and shrubs had the greatest variation in specific root length (SRL) (55.55 %–71.46 %) and the least variation in root carbon content (RCC), with all values below 5 %. Root diameter (RD), RCC and root carbon to nitrogen ratio (RC/N) for 1–3 order roots, and specific root surface area (SRA) for second-order roots were greater for trees compared with those for shrubs. Only the first-order root trait exhibited significant differences. Principal component (PC) 1 and PC2 of 1–3 order roots cumulatively explained 72.10 %, 71.60 %, and 67.20 % of the variance, respectively. Each sequence axis of first and second-order roots was positively correlated with SRL and SRA, and the third-order roots were negatively correlated with these traits. The soil total potassium content (STKC), soil total phosphorus content (STPC) and soil rapidly available phosphorus content (SAPC), had the greatest effect on plant root morphological traits. The findings suggest that in this study area, trees are more likely to adopt resource-conserving survival strategies, whereas shrubs tend to adopt resource-acquiring strategies.

## 1 Introduction

Fine roots, as the primary organs responsible for water and nutrient uptake in trees, have several advantages, including a large surface area, high metabolic activity (physiological and physical) and a high rate of mycorrhizal infestation [1], 2]. These roots play a critical role in key ecosystem processes, such as community dynamics, soil formation, carbon accumulation, and nutrient cycling [3]–6]. Absorptive roots are fine roots with a relatively rapid turnover rate, primarily responsible for resource uptake, and consist mainly of 1–3 order roots [7]. The survival and propagation of plants depend upon their ability to adapt to specific environmental conditions through the adjustment of a series of functional traits, including morphological, physiological, and chemical adaptations [8]–12]. In habitats with limited resources or subject to abiotic stresses (such as nutrient-poor soils, drought, or extreme temperatures), this adaptive differentiation is particularly pronounced and frequently unfolds along an “acquisition-conservation” resource economy spectrum [13]. A high specific root length (SRL) may result from the increased soil exploration and uptake enabled by long thin roots [14]–17]. Conversely, high values of root tissue density and root diameter (RTD and RD) are thought to maximise longevity while minimizing cellular activity and maintenance respiration needs [18]–20]. High RTD may enhance longevity by increasing resistance to herbivory and drought stress through cell-wall thickening and lignification [21].

The root characteristics of different forms of plants vary to a certain extent [22]. Du et al.’s research on the root morphology characteristics of woody plants in subtropical natural evergreen broad-leaved forests revealed the significantly greater and smaller RD and SRL of trees than those of shrubs, respectively; the trees in the Yanhe River Basin have a high carbon content and strong defence capacity, the fine roots of evergreen species follow a more conservative strategy, and those of deciduous species adopt a more rapid growth strategy; these adaptive strategies for plant root traits are inseparable from the plant’s root economics spectrum (RES) [23]–25]. At present, the RES ties together demographic factors of surface absorptive roots into two groups of associated traits [13], 26]. Acquisitive fine root systems are expected to have higher root nitrogen concentration (RNC) and SRL, which result in quick returns on investment and high turnover rates. Conversely, absorptive roots with greater average RD and RTD have greater upfront costs and slower returns on investment but are associated with lower maintenance needs, improved stress tolerance and longer lifespans [21], 27], 28]. Variations in the fine root characteristics of different life form plants give rise to distinct structural features, which fully reflect the adaptive strategies of plants towards their habitats.

Lava platforms are unique habitats formed after volcanic eruptions. Jingpo Lake, as China’s largest volcanic lava-dammed lake, boasts unique geological features and a rich cultural heritage, characterised by its flat terrain. The geological structure primarily consists of granite, perlite and black basalt. Soil resources are relatively scarce, and the ecological environment presents challenging conditions. [29]. Huang et al. demonstrated that herbaceous plants dominate the vegetation on the Wudalianchi lava platform, outperforming other vegetation types. This finding may stem from the harsh environmental conditions posing significant challenges to the establishment, growth and survival of woody plants. Consequently, the diversity, abundance and distribution of woody plant groups – including trees and shrubs – are constrained, which impedes the pace of vegetation recovery and succession on the lava platform [30]–32]. A survey revealed that the study area harbours a limited number of woody plant species, with only four shrub species present. This condition severely constrains the development of woody plant biodiversity within the region. The adaptive characteristics enabling woody plants to thrive in this heterogeneous habitat warrant further investigation. Thus, this study focused on the absorbent roots (1–3 order roots) of 16 woody plant species on the Jingpo Lake lava platform and measured the morphological and chemical characteristics of each root order. We tested two hypotheses: (1) variation in root system traits among plants on the Jingpo Lake lava platform is unaffected by life form or root sequence; tree species adopt a resource-conserving survival strategy, whereas shrub species employ an acquisition-oriented survival strategy; (2) soil factors, particularly nitrogen and phosphorus content, may considerably influence the root traits of tree and shrub species in the lava platform. This study investigated the interrelationships among various root sequence traits in fine roots and their RES characteristics, aiming to validate how different life forms (trees and shrubs) exhibit differentiated adaptive strategies along this spectrum within the harsh lava platform environment. It holds scientific importance for maintaining the ecological stability of lava platform ecosystems and promoting ecosystem restoration.

## 2 Materials and methods

### 2.1 Study sites

**Figure 1:**
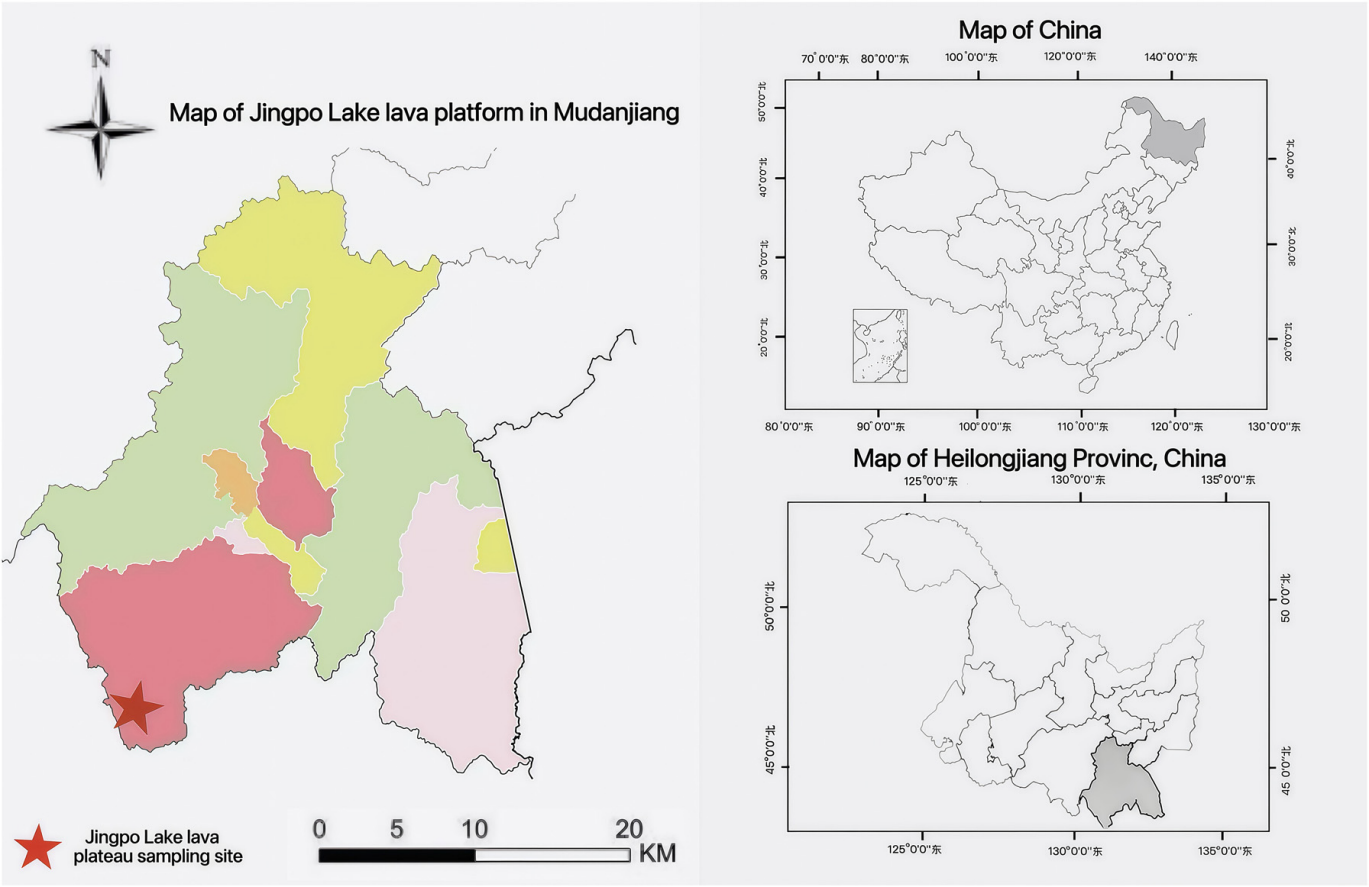
Map of Jingpo Lake lava platform in Mudanjiang.

Jingpo Lake Geopark (128°30′–129°11′ E, 43°34′–44°17′ N) is located between the mountain ranges of Zhangguangcailing and Laoyeling and belongs to the low mountain hilly landscape (Figure 1). The site has a changeable climate in spring and autumn, with a large temperature difference between morning and evening in summer and cold and dry in winter. The average annual precipitation is 506.4 mm, the average annual temperature reaches 3.6 °C, and the annual temperature difference is as high as 38 °C-48 °C. The lava platform is located in the northern part of Jingpo Lake, with a flat terrain and average soil physicochemical properties (*n* = 16) as follows: pH 6.38; total carbon, 141.34 g·kg^−1^; total nitrogen, 1.86 g·kg^−1^; total phosphorus, 0.62 g·kg^−1^; total potassium, 28.67 g·kg^−1^; effective nitrogen, 71.54 mg·kg^−1^; effective phosphorus, 52.35 mg·kg^−1^; effective potassium, 58.48 mg·kg^−1^.

### 2.2 Sampling collection

In this study, we selected 16 species of dominant woody plants in the Jingpo Lake lava platform, including 12 species of trees and four species of shrubs, belonging to nine families and 14 genera. The plant lists are shown in Table 1. Sampling was carried out in August 2023, and three fixed sample plots of 100 m × 100 m were divided among the sampling plots, with each spaced at an interval of more than 50 m. Plant roots and soil were collected in each sample plot. In each sampling site, three plants exhibiting vigorous growth and free from disease or pest infestation were selected for each species. Within a radius of 0.5–1 m from each plant, the main root was selected in three directions at the base of the extended trunk, and 3–4 complete branches containing 1–3 levels of roots were selected from each plant.

**Table 1:** Family, genus and life forms of 16 species.

Number	Species	Symbol	Family	Genus	Life form
1	*Betula platyphylla*	BP	Betulaceae	Betula	Tree
2	*Betula costata*	BC	Betulaceae	Betula	Tree
3	*Acer pictum* subsp.	AP	Aceraceae	Acer	Tree
4	*Acer negundo* L.	AN	Aceraceae	Acer	Tree
5	*Populus* L.	PL	Salicaceae	Populus	Tree
6	*Salix raddeana*	SR	Salicaceae	Salix	Tree
7	*Prunus padus* L.	PP	Rosaceae	Prunus	Tree
8	*Prunus sibirica* L.	PS	Rosaceae	Prunus	Tree
9	*Albizia kalkora*	AK	Fabaceae	Albizia	Tree
10	*Ulmus pumila* L.	UP	Ulmaceae	Ulmus	Tree
11	*Quercus mongolica*	QM	Fagaceae	Quercus	Tree
12	*Phellodendron amurense*	PA	Rutaceae	Phellodendron	Tree
13	*Sorbaria sorbifolia*	SS	Rosaceae	Sorbaria	Shrub
14	*Rosa acicularis*	RA	Rosaceae	Rosa	Shrub
15	*Amorpha fruticosa*	AF	Fabaceae	Amorpha	Shrub
16	*Flueggea suffruticosa*	FS	Phyllanthaceae	Flueggea	Shrub

Around each plant, three soil samples collected from 0–40 cm depths were composited into one replicate for the species. The soil was brought back to the laboratory, air dried, ground and sieved through a 0.15 mm sieve. The sieved soil was used for nutrient content determination. The collected plant roots were placed in self-sealing bags, brought back to the laboratory, rinsed with distilled water, dried and labelled.

### 2.3 Determination of functional traits

The fresh root was classified into 1–3 orders of fine roots (three plants one species) using the root hierarchy classification method described by Pregitzer. In addition, the roots were scanned with Espon scanner, after which they were classified into sequential classes, placed in envelope bags and dried for 48–72 h to constant weight [33]. The dried root samples were ground and sieved through a 100-mesh sieve for the determination of carbon and nitrogen chemical traits. Roots were analysed using the root image analysis software Win RHIZO (Pro 2005b) to directly obtain the total root length, RD, root surface area and root volume. The SRL, RTD and SRA were also measured. An elemental analyser (ECS4010, Costech, Milan, Italy) was used to determine root carbon content (RCC) and RNC in the fine root samples and to calculate root carbon/nitrogen (RC/N). The descriptions and calculation methods are shown in Table 2.

**Table 2:** Description and calculation methods of each trait.

Traits	Symbol	Unit	Calculation	Description
Root diameter	RD	mm	/	Refers to the segment on the root cross section that passes through the center of the circle and both ends are on the circumference
Specific root length	SRL	m·g^−1^	Root length/root dry weight	Refers to the root length of fine root unit weight
Root tissue density	RTD	g·cm^−3^	Root dry weight/root volume	Ratio of root dry weight to root volume
Specific root surface area	SRA	cm^2^·g^−1^	Root surface area/root dry weight	Ratio of finger root surface area to dry weight
Root carbon content	RCC	mg·g^−1^	/	Carbon content in root
Root nitrogen content	RNC	mg·g^−1^	/	Nitrogen content in root
Root C/N	RC/N	NA	Total carbon content/total nitrogen content	Ratio of root carbon content to root nitrogen content

### 2.4 Determination of soil physical and chemical properties

Soil nutrient determination was performed in accordance with soil agronomic analysis [34]. Soil pH was measured using the potentiometric method; total nitrogen content (STNC), the semi-micro Kjeldahl method, ammonium nitrogen (SANC), the indophenol blue colorimetric method; total phosphorus content (STPC), the sulfuric acid–perchloric acid digestion method. NaHCO_3_ extraction–molybdenum antimony colorimetric method was used to determine soil available phosphorus (SAPC); acetic acid–nitric acid powder colorimetric method, soil nitrate nitrogen (SNNC); flame photometric method, total potassium content (STKC).

### 2.5 Data analysis

For all measured variables, normality of distribution was tested using the Kolmogorov–Smirnov test (*P* = 0.05), and homogeneity of error variance was verified via Levene’s test (*P* = 0.05). One-way analysis of variance (ANOVA) was performed to examine significant differences in all root morphological traits between tree (*n* = 12) and shrub (*n* = 4) species, with Tukey’s test used for post-hoc multiple comparisons of means. Descriptive statistics, including the mean, standard error, and coefficient of variation, were calculated for each root morphological trait index of the two functional groups (trees and shrubs). Pearson correlation analysis was conducted to explore the associations among root morphological traits within each functional group (trees and shrubs separately). Principal component analysis (PCA) was performed based on standardized data to characterize the patterns of variation in root traits across all species (12 trees and four shrubs). Redundancy analysis (RDA) was used to quantify the relationship between root traits and soil factors, while a Mantel test with 999 permutations via Monte Carlo simulation was supplementary to verify the correlation between the root trait matrix and soil factor matrix. All statistical analyses were performed using Microsoft Excel 2007 and SPSS software (Version 19.0; SPSS Inc., Chicago, IL, USA), with graphs generated using ORIGIN and Canoco 5.

## 3 Results

### 3.1 Coefficients of variation of morphological and chemical traits of absorbing roots of different life forms

As shown in Table 3, among the different root orders of trees, morphological traits exhibited the widest range of variation for SRL and the narrowest range for RD, and chemical traits showed the highest coefficient of variation for RNC and the lowest for RCC. As root order increased, the coefficients of variation for all morphological traits exhibited irregular changes. The coefficients of variation for RD and RTD remained within a low-intensity variation range, and those for SRL and SRA fell within a high-intensity variation range. In shrubs, the ranges of variation for morphological and chemical traits under different root orders are the same as those in trees. RD remained within the low-intensity variation range; the coefficient of variation for SRL was always greater than 50 % was generally fell within the high-intensity variation range. The coefficient of variation for RTD in roots of grades 1–3 increased with increasing root order, except for the first-order roots. All the other root orders belonged to the high-intensity variation range; the coefficient of variation for SRA only belonged to the high-intensity variation range in the third-order roots.

**Table 3:** Variation coefficient of root morphological and chemical traits for different life forms of 16 plants.

Species	Root order	Root trait	Mean ± SD	Max	Min	CV (%)
Tree	First-order root	RD (mm)	0.13 ± 0.03	0.17	0.08	23.07
		SRL (m·g^−1^)	89.51 ± 49.72	153.83	20.84	55.55
		RTD (g·cm^−3^)	1.25 ± 0.50	2.04	0.37	40.00
		SRA (cm^2^·g^−1^)	300.26 ± 130.59	575.23	106.52	43.49
	Second-order root	RD (mm)	0.15 ± 0.04	0.19	0.10	26.67
		SRL (m·g^−1^)	58.73 ± 36.64	117.58	13.96	62.39
		RTD (g·cm^−3^)	1.68 ± 0.64	3.43	1.04	38.10
		SRA (cm^2^·g^−1^)	212.89 ± 84.89	336.26	81.32	39.88
	Third-order root	RD (mm)	0.22 ± 0.04	0.28	0.14	18.18
		SRL (m·g^−1^)	22.44 ± 16.02	54.58	4.43	71.39
		RTD (g·cm^−3^)	2.19 ± 0.90	4.27	1.48	41.10
		SRA (cm^2^·g^−1^)	129.05 ± 64.75	235.91	43.84	50.17
	RNC (mg·g^−1^)		16.79 ± 1.64	31.52	10.88	33.89
	RCC (mg·g^−1^)		455.87 ± 6.04	491.71	412.77	4.59
	RC/N		29.40 ± 2.22	40.05	14.66	26.19
Shrub	First-order root	RD (mm)	0.11 ± 0.03	0.13	0.07	27.27
		SRL (m·g^−1^)	128.64 ± 91.92	255.76	36.79	71.46
		RTD (g·cm^−3^)	1.20 ± 0.43	1.83	0.90	35.83
		SRA (cm^2^·g^−1^)	373.45 ± 175.86	575.64	147.42	47.09
	Second-order root	RD (mm)	0.14 ± 0.03	0.18	0.10	21.43
		SRL (m·g^−1^)	54.29 ± 31.25	89.17	13.88	57.56
		RTD (g·cm^−3^)	1.81 ± 1.04	3.36	1.12	57.46
		SRA (cm^2^·g^−1^)	207.31 ± 92.04	278.30	74.52	44.40
	Third-order root	RD (mm)	0.22 ± 0.07	0.31	0.16	31.82
		SRL (m·g^−1^)	25.95 ± 18.36	43.33	3.75	70.75
		RTD (g·cm^−3^)	2.15 ± 1.35	4.14	1.20	62.79
		SRA (cm^2^·g^−1^)	134.05 ± 72.38	198.38	37.5	53.99
	RNC (mg·g^−1^)		20.74 ± 3.30	26.92	12.72	31.82
	RCC (mg·g^−1^)		453.74 ± 6.30	470.31	443.08	2.78
	RC/N		23.87 ± 4.21	34.84	16.53	35.27

### 3.2 Differences in morphology and chemical traits of absorbing roots in different life forms of plants

**Figure 2:**
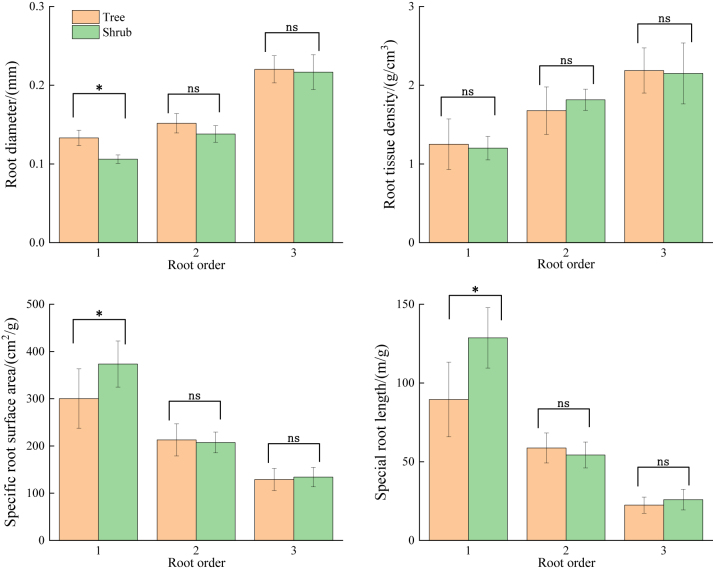
Differences in morphological traits of 1–3 order roots in different life forms of plants. Note: Different lowercase letters indicate significant differences between plants (*P* < 0.05).

**Figure 3:**
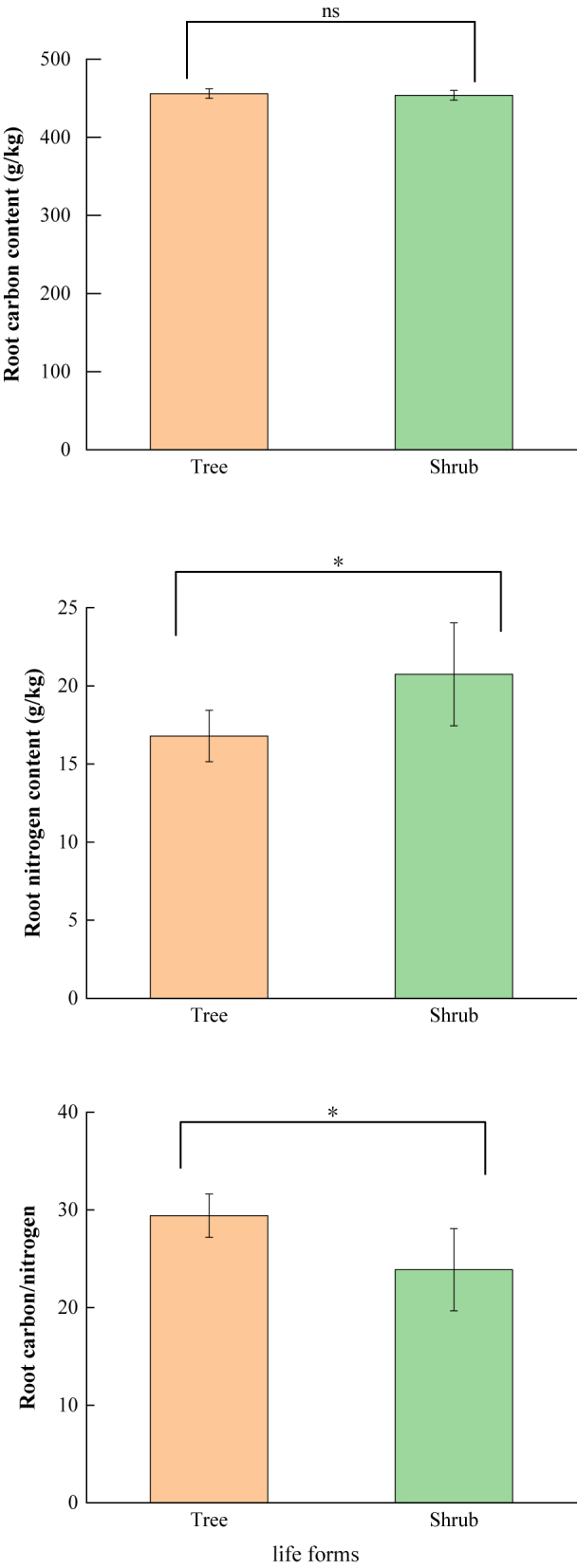
Chemical properties of fine roots of plants with different life forms. Note: different lowercase letters indicate significant differences between plants (*P* < 0.05).

The analysis of morphological traits across different plant life forms revealed that RD and RTD increased, while SRL and SRA decreased with increasing root order. In the first-order roots, RD and RTD were higher in trees than in shrubs, while SRL and SRA were lower in trees compared with shrubs. Differences in all traits, except for RTD, were significant (*P* < 0.05). In second-order roots, RD, SRA and SRL were greater in trees than in shrubs, and RTD was greater in shrubs than in trees, with no significant differences among traits (*P* > 0.05). The pattern in third-order roots mirrored that of first-order roots, although no significant differences were found among the traits (*P* > 0.05) (Figure 2). The analysis of chemical traits revealed a significant difference in RNC and RC/N between different life forms (*P* < 0.05). Specifically, trees had higher RCC and RC/N, and shrubs had higher RNC (Figure 3).

### 3.3 Linkages between absorbing root morphology and chemical traits in woody plants of different life forms

**Figure 4:**
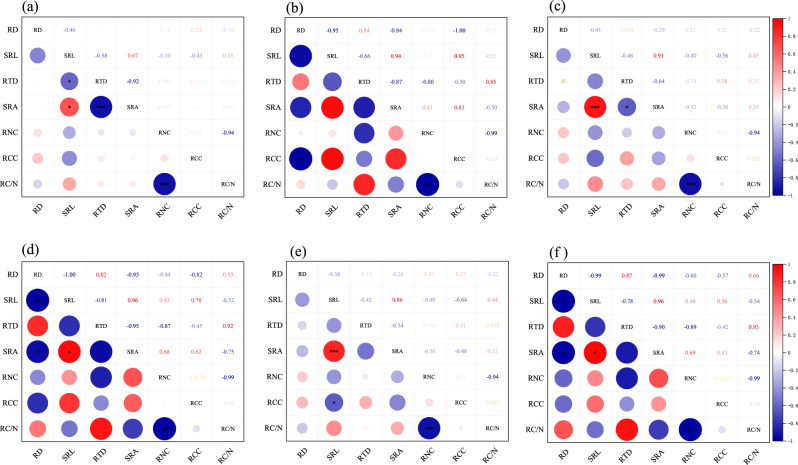
Correlation analysis of root traits of tree and shrub. Note: *P ≤ 0.05 **P ≤ 0.01 ***P ≤ 0.001; (a) correlation of first-order root of trees; (b) correlation of first-order root of shrubs; (c) correlation of second-order root of trees; (d) correlation of second-order root of shrubs; (e) correlation of third-order root of trees; (f) correlation of third-order root of shrubs; red indicates positive correlation, blue indicates negative correlation; see Table 2 for abbreviations.

Correlation analysis revealed that in the roots of tree plants (1–3 order roots), SRL and RTD showed a highly significant positive correlation and a significant negative correlation with SRA, respectively. In addition, RNC was highly negatively correlated with RC/N. Among these traits, SRL displayed a significant negative correlation with RTD in the first-order roots and a significant negative correlation with RCC in the second- and third-order roots. In the roots of shrub plants of 1–3 order roots, RD showed a highly significant negative correlation with SRL, RNC and RC/N, and SRL displayed significant positive correlation with SRA. The first-order roots showed a highly significant negative correlation with RCC, and SRL revealed a significant positive correlation with RCC. the second-order roots presented a significant negative correlation with RTD and SRA; the second- and third-order roots showed a significant RD and SRA. The RD of the second- and third-order roots mainfested a significant negative correlation with SRA, and RTD displayed a significant positive correlation with RC/N (Figure 4).

### 3.4 PCA of root morphology and chemical traits of 16 plant species

**Figure 5:**
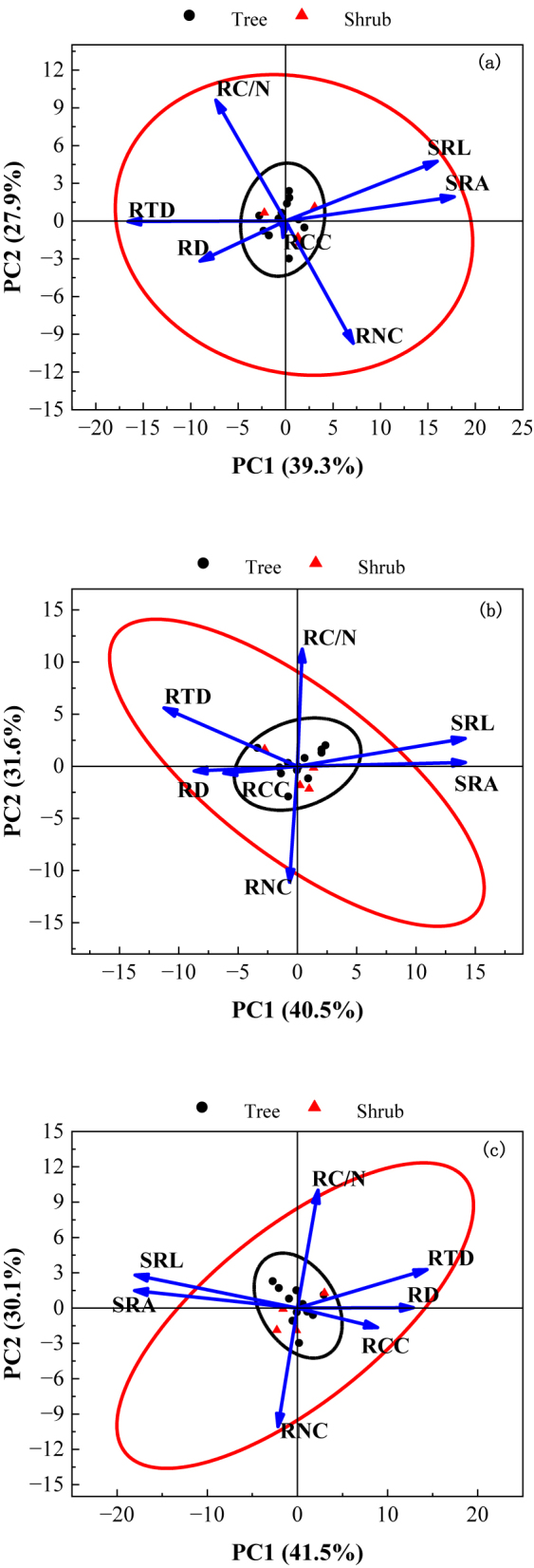
PCA of morphology and chemical traits in different root sequences of different life forms. Note: a: first-order root; b: second-order root; c: third-order root.

The results of PCA (Figure 5) showed that PC1 and PC2 of the 1–3 order roots collectively explained 72.10 %, 71.60 %, and 67.20 % of the variance in root morphology and chemical traits, respectively, with PC1 contributing more than PC2 in all cases. In the first-order roots, the PC1 axis, defined by SRA, SRL, RTD and RD, explained 39.3 % of the variance, and the PC2 axis, defined by the RC/N, RNC and RCC, explained 27.9 %. For the second-order roots, the PC1 axis, defined by SRL, SRA, RTD, RD and RCC, explained 40.5 % of the variance, and the PC2 axis, defined by RC/N and RNC, explained 31.6 %. In the third-order roots, the PC1 axis, defined by RTD, RD, RCC, SRL and SRA, explained 41.5 % of the variance, and the PC2 axis, defined by RC/N and RNC, explained 30.1 %. The first- and second-order roots of the 16 woody plants were mostly distributed in the right half of the PC1 axis and characterised by large SRL and SRA, which suggest a strong capacity for water and nutrient uptake. By contrast, the third-order roots were mostly distributed in the left half of the PC1 axis and characterised by larger RD, RTD and higher RCC. This distribution indicates that the first- and second-order absorptive roots, the primary axis (PC1) represented an acquisition-conservation spectrum, with high SRL/SRA on one end. For the third-order roots, this axis flipped, indicating these roots are transitioning to a more conservative, transport/storage role.

### 3.5 RDA of morphological and chemical traits of 16 plant roots with soil traits

**Figure 6:**
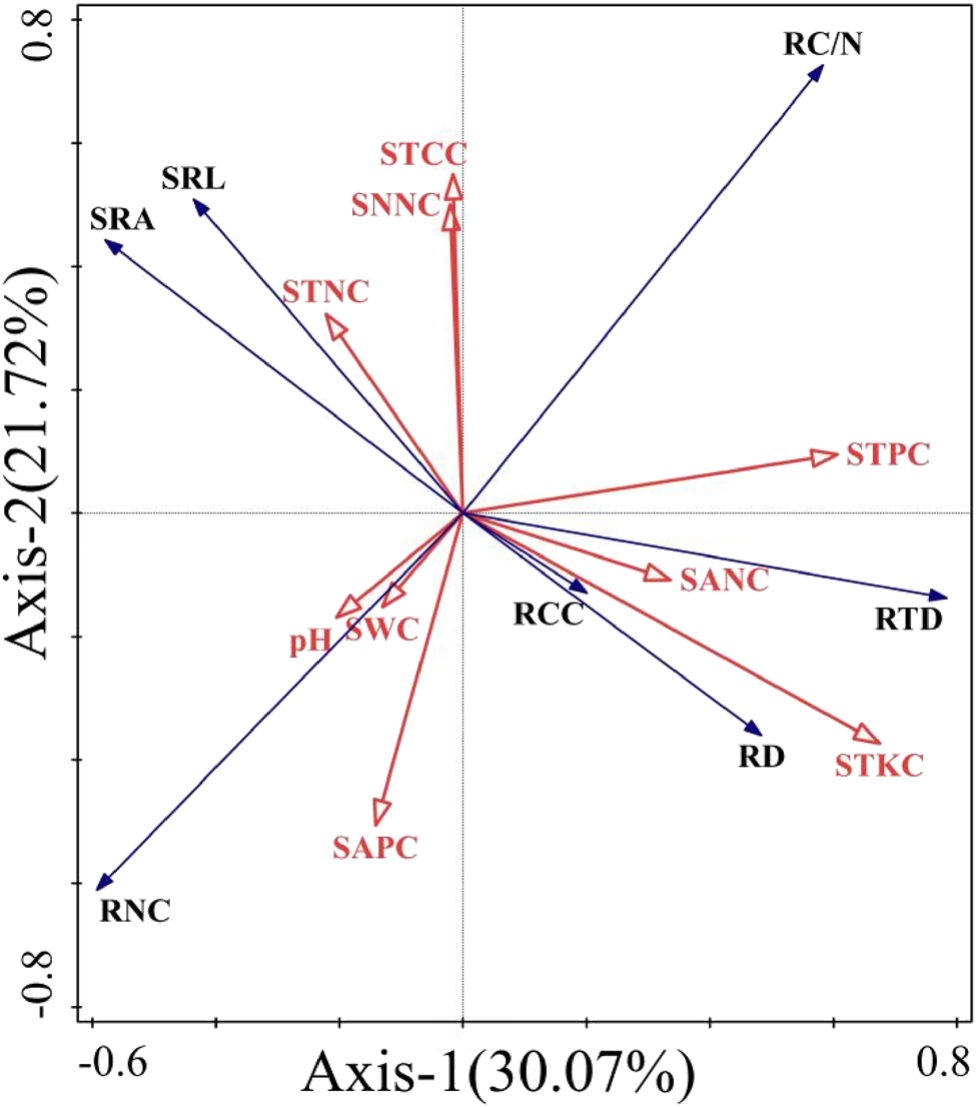
RDA of plant root traits and soil physicochemical factors. Note: The red arrow represents the soil factor: SWC, soil water content; pH, potential of hydrogen; STCC, soil total carbon content; STNC, soil total nitrogen content; STKC, soil total potassium content; STPC, soil total phosphorus content; SANC, soil ammonium nitrogen content; SNNC, soil nitrate nitrogen content; SAPC, soil rapidly available phosphorus content. The blue arrows represent various traits of plant root: RD, root diameter; SRL, specific root length; RTD, root tissue density; SRA, specific root surface area; RCC, root carbon content; RNC, root nitrogen content; RC/N, root carbon/nitrogen.

RDA (Figure 6) revealed that the first- and second-order axes collectively explained 51.79 % of the relationship between the morphological traits of absorptive roots (1–3 order roots) of common woody plants in lava platforms and soil environmental factors. Among environmental variables, STKC, STPC and SAPC had the most significant influence on plant root morphological traits, and the contribution rate of them were 24.2 %, 17.8 %, and 13.9 %, respectively (*P* < 0.05) (Table 4). The correlations among root functional traits and soil nutrients were ranked as follows: STKC > STPC > SAPC > STCC > STNC > SNNC > pH > SANC > SWC. Specifically, STPC, SANC and STKC were strongly positively correlated with RTD, RNC, RD, and RC/N and strongly negatively correlated with SRL, SRA and RCC. Conversely, STCC, SNNC and STNC were positively correlated with SRA, SRL and RC/N SWC, SAPC and pH were positively correlated with RCC.

**Table 4:** Mantel test results for the corelation between relative abundance of root traits and soil.

Soil variables	STNC	pH	SWC	STPC	STKC	SNNC	SANC	SAPC	STCC
R^2^	0.079	0.056	0.021	0.178	0.242	0.068	0.048	0.170	0.139
*P*	0.258	0.398	0.825	**0.047**	**0.022**	0.293	0.458	**0.044**	0.060

Note: Data in bold indicates statistically significant differences.

## 4 Discussion

### 4.1 Characteristics of variation in morphological and chemical traits of roots of different life forms of plants

The degree of variation in functional traits reflects a plant’s adaptive strategy to its environment, and the extent of this variation is influenced by various factors, such as climate, topography and terrain [35]. Lava platform soils have low developmental levels, and their physical and chemical properties (such as nutrient availability, porosity, and water permeability) exhibit a high spatial heterogeneity at the microscale, this heterogeneity may force plants to adapt to the environment by adjusting their root morphology [36]. In this study, all root traits of the 16 woody plant species exhibited varying degrees of variation. In trees and shrubs, the coefficient of variation for SRL was the highest across all root orders, and that for RCC was the lowest, which substantiates our first hypothesis. Hodge experimentally confirmed that plants in soils with uneven nutrient distribution prioritise increasing the proliferation rate of local root systems, forming a ‘foraging strategy’. This elevated SRL variation intensity may represent a mechanism underpinning the enhanced root morphological plasticity observed in lava platform vegetation [37]. Yang studied 25 common desert plants in the northwestern arid region and found that SRL and SRA exhibited the highest overall variability, and RCC and RD showed the lowest variability. This finding aligns with the results for trees in this study. RCC represents the most stable variable along the ‘investment-profit’ strategy axis, and it is potentially constrained by the conservative strategy of plant carbon metabolism [38]. Roumet et al.’s research indicated that RCC typically varies less among species than morphological traits, as it is more directly associated with plant growth, development and stress resistance functions [39]. In the highly stressed environment of the Jingpo Lake lava platform, maintaining the stability of RCC may have adaptive significance in coping with the nutrient-poor and structurally uneven conditions of the lava platform.

### 4.2 Survival strategies of different life form plants on lava platforms

The RD and SRL of plant roots reflect a plant’s adaptation to the environment and its efficiency in nutrient utilisation, and RTD and SRA are often associated with the plant’s root extension ability and resource acquisition capacity [40], 41]. In this study, shrubs had smaller RD and RTD values but larger SRL and SRA values, which demonstrates that the root systems of shrubs are more dispersed and extensive; this outcome increasde the surface area available for nutrient uptake from the soil and enhanced nutrient utilisation efficiency; shrubs utilise water and nutrients more efficiently than trees, representing a resource-acquisition-oriented survival strategy [42]. By contrast, the larger RD values in trees are typically associated with thicker bark and xylem layers, which facilitates mycorrhizal colonisation for nutrient acquisition. In addition, they allocate more carbon and nutrients to root development, which results in longer lifespans, representing a resource-conservative survival strategy; this finding confirms our first hypothesis, this may be attributed to shrubs’ smaller size, shorter lifespan, and need for rapid growth to establish in open areas. Trees, as long-lived dominants, invest in persistence [13], 43]–45]. Shrubs have larger SRA in their absorptive roots, which increases the root system’s contact area with the soil and thereby enhances the efficiency of water and nutrient exchange between plants and soil. From the perspective of different root orders, only the RD, SRL and SRA of primary roots in trees and shrubs show significant differences, which may be closely related to the fact that lower-order roots primarily function for absorption, lack secondary growth processes, have short lifespans and exhibit intense metabolic activity.

Trees and shrubs, as two common plant growth forms, exhibit considerable differences in their growth environments, life forms and ecological functions, which result in distinct variations in their carbon and nitrogen content [46], 47]. In the two life form plants studied here, the RCC and RN/C values of trees were higher than those of shrubs, and only RNC and RN/C showed significant differences, consistent with previous research findings [48]. These results were observed may be because trees typically have greater height and growth rates, with more developed root systems. Their deeper root systems enable them to more effectively access carbon and nitrogen resources in the soil, and this growth form often results in higher carbon and nitrogen content in trees [49], 50]. By contrast, shrubs are generally shorter in stature and have relatively shallow and dispersed root systems, which results in lower carbon and nitrogen resource acquisition capacity [51]. These factors lead to differences in carbon and nitrogen allocation between trees and shrubs, thereby influencing their growth and competitive capabilities within ecosystems [52]. Given that the two life forms examined in this study differ in species richness (12 trees and four shrubs), and that these four shrub species represent the only successfully established tree species in the local environment, their adaptive strategies hold significant research value and are representative of plant survival strategies in this region. However, whether these findings can be extrapolated to broader ecological contexts, serving as universal strategies for tree and shrub life forms, warrants further investigation.

In general, the root structure of plants shows a close linkage to their growth environment and ecological adaptability. In nutrient-rich environments, plants tend to invest more resources in their underground parts to acquire more carbon and nitrogen, which supports their growth and reproduction [53]. However, in nutrient-poor or water-stressed environments, plants may expand the contact area between their root systems and the soil to enhance their survival competitiveness. Therefore, root structure not only reflects a plant’s adaptive response to growth but also embodies its carbon-nitrogen allocation strategy under different environmental conditions, with the root branching ratio determining root structure; complex fine root branching configurations can increase fine root surface area, thereby enhancing absorption efficiency while also increasing root system construction costs [54].

### 4.3 Trade-offs among plant traits on lava platforms

Plant growth is influenced by various factors, resulting in relationships among different root traits that reflect plants’ adaptation strategies to diverse environments. Bergmann et al. and Ding et al. indicated that variation in fine root traits can be divided into two main dimensions, known as the RES [45], 55]. This study revealed that as the root order changes, SRL and SRA in trees always showed a positive correlation, and RTD and SRA exhibited a negative correlation. In shrubs, SRL and SRA consistently showed a positive correlation, and RD and SRL had a significant negative correlation, which reflects the plants’ convergent evolution strategies. Given that fine root N is typically understood to reflect enzymatic capacity, high values are predicted to represent a quick return on investment strategy. Here, RNC showed a positive correlation with SRL, which demonstrated that fine roots with a greater investment in length growth and possibly soil exploration likely also have more enzymatic capacity. The shrubs at this end of the spectrum, owing to their highly dispersed root systems and growth strategy centred on seeking out additional resources, may have enhanced root decomposition under the nutrient-poor soil conditions of the terraces. This condition has rendered their high resource acquisition strategy more effective. The RNC of trees was negatively correlated with SRL. These differing correlations between trees and shrubs indicate distinct resource acquisition and adaptation strategies. As RNC shows a is close correlation with the respiratory rate of plant fine roots, the conservative end of the lineage indicates that lava plateau trees, in adapting to their environment, may exhibit lower metabolic and maintenance respiration in their fine roots, which entail higher construction costs [56]. This finding further substantiates our first hypothesis.

Wen studied 21 tree species in the Maoershan Experimental Forest Farm in Northeast China [57]. By contrast, the 1–3 order roots of trees in this study area exhibited smaller RD and larger RTD values. Compared with those observed in the studies by Sun and Xu, the 1–3 order roots of woody plants in this study area also showed smaller RD and larger RTD values [40], 41]. Based on the PCA results, woody plants on the Jingpo Lake lava platform generally adopt a conservative survival strategy, characterised by small RD and large RTD. The expressions of plant functional traits varied across different environments, which reflects ecological strategies that enhance feasibility and adaptability under specific conditions. The research by Freschet et al. on the RES indicates that roots with highe SRA and N and P content typically acquire resources more efficiently but have short lifespans [58]. Conversely, roots with lower SRA and nutrient content tend to have longer lifespans, although this relationship may be influenced by specific root structures [59].

Soil factors play a crucial role in determining plant trait variation at a localised scale. The heterogeneity of plant traits is positively correlated with soil heterogeneity. In this study, STKC, STPC and SAPC had the greatest influence on plant root morphological traits, which demonstrates our second hypothesis, this may be attributed to the strong phosphorus immobilisation within the soil of the Jingpo Lake lava platform, coupled with potential potassium leaching or immobilisation, collectively constituting key nutrient limiting factors. This directly drives local woody plants to evolve distinct root functional traits, differentiating into “acquisitive” (shrubs) and “conservative” (trees) strategies to cope with prolonged nutrient stress. Moreover, SRL showed a significant positive correlation with STCC and STNC, which indicates that shrub plants in this area have higher nutrient availability and faster root turnover rates. Owing to the intricate nutrient cycling processes established between vegetation and soil, ecosystems rely on these nutrient exchange mechanisms to optimally utilise various nutrient elements for natural restoration. Based on an analysis of the adaptive characteristics of tree and shrub species on lava platforms towards soil conditions, we contend that ecosystem restoration and management should prioritise the conservation and rehabilitation of plant ecosystems. This approach necessitates minimising anthropogenic interference and disturbance wherever possible, thereby preserving the stability and sustainability of the ecosystem [60].

## 5 Conclusions

In this study, we analysed the differences in morphological and chemical traits of absorptive roots in tree and shrub species by examining the root systems of 16 woody plant species from the Jingpo Lake lava platform. This analysis aimed to explore the adaptive mechanisms of different life forms to the heterogeneous habitats of the lava platform. In the study area, shrubs, with thinner first order roots and higher RNC, are at the “acquisitive” end of the spectrum, favoring rapid resource capture in a competitive, nutrient-poor environment. Trees, with thicker roots and higher C/N, are at the “conservative” end, investing in durable structures for long-term survival. From the perspective of the relationship between plants and soil, plant root uptake in the study area was primarily influenced by STKC and STPC. These findings provide valuable insights into plant root resource acquisition and adaptation strategies. Moreover, the research on the RES holds significant implications for ecological conservation and plant restoration in this region. However, for the establishment of more precise ecological restoration strategies, further investigation of the spatial heterogeneity of ecosystems at the regional scale is required.
